# Comments on: Effects of Wi-Fi (2.45 GHz) Exposure on Apoptosis, Sperm Parameters and Testicular Histomorphometry in Rats: A Time Course Study

**DOI:** 10.22074/cellj.2016.3848

**Published:** 2016-01-17

**Authors:** Seyed Mohammad Javad Mortazavi, Hossein Mozdarani

**Affiliations:** 1 Ionizing and Non-ionizing Radiation Protection Research Center (INIRPRC), Shiraz University of Medical Sciences, Shiraz, Iran; 2 Medical Physics and Medical Engineering Department, School of Medicine, Shiraz University of Medical Sciences, Shiraz, Iran; 3 Department of Medical Genetics, School of Medicine, Tarbiat Modares University, Tehran, Iran

We read with great interest an article by Shokri et al. entitled "Effects of Wi-Fi (2.45 GH z) exposure on apoptosis, sperm parameters and testicular histomorphometry in rats: a time course study" that is published in the latest issue of the Cell Journal (Vol.17, 2015: 322-331). In this article, Shokri et al. have presented their findings obtained in an experiment on an animal model. These researchers exposed rats to the 2.45 GHz radiation in a chamber with two Wi-Fi antennas on opposite walls of a box. The exposed animals in this study showed a decrease in sperm parameters. We have previously shown that exposure to electromagnetic fields generated by Wi-Fi routers or mobile phone jammers can adversely affect the sperm quality (1-3). The paper published by Shokri et al. is seriously flawed. The first major shortcoming of this paper is its exposure geometry. The authors stated that their exposure system was "a chamber (180 cm×80 cm×70 cm), designed for whole-body exposure of free-moving rats to a Wi-Fi signal. Two Wi-Fi antennas (NanoStation Loco M2, 2.45 GHz, 8.5 dBi, Ubiquiti Networks, Inc. USA) were placed at the center of two sides of the chamber". It should be noted that in this case, the power density can be calculated using the below equation:

$$S=P•G/4\pi R^{2}$$

Where

S=Power density
P=Power input to antenna
G=Antenna gain

In this light, the geometry used in the study of Shokri et al. makes a very inhomogeneous distribution of power densities. The second shortcoming comes from this point that the authors claimed that their study was performed on a basis that could not affect the hormonal balance "A previous study applied a restrainer to fix space between antenna and rat. Since it was a stressful condition that could probably affect hormonal balance of animals, we tried to assess the effect of radiation on the free moving animals". However, these authors only had a control group and did not use a sham-exposed group to control the animals’s stress and its subsequent hormonal changes. Furthermore, another shortcoming comes from this point that "NanoStation Loco M2" is not a standard Wi-Fi router. As manufacturer reports this device is a compact outdoor communication unit that can be used for devices such as cameras "NanoStation Loco M2 is a compact outdoor unit which includes 2×8 dBi antenna (MIMO) for the 2.4 GHz band”. Therefore, it is misleading to claim that in this study the effects of Wi-Fi exposure on apoptosis are investigated and the title of this paper is indeed incorrect "Effects of Wi-Fi (2.45 GH z) exposure on apoptosis, sperm parameters and testicular histomorphometry in rats”. We hope that these comments are helpful to make more reliable results in the future.

## Reply of the Author

In this project we read your great papers. By the way, we designed a system close to human living medium. Therefore, we did not use a restrainer. And, we used plastic cage in equal size of standard rat cage and animals move completely free without any stress and food and water were available. And, the hormonal assay was not our criteria. Therefore, it is not necessary to use sham operated animals.

Moreover, we put the the cage randomly into the box that had antenna same as human. For example, a cage was close the antenna today and may be it will in the center of box and far from the antenna tomorrow. Therefore, space between antenna and animal was not our criteria. Therefore, we did not measure the geometry.

Finally, we had medical engineer consultant and before buying these antenna consulted him and sent an email the company and they confirmed that these antenna are equal with Wi-Fi (2.45 GHz). Bye the way, I will ask him and company again and follow this issue.

The best criterion was the time exposure (1 hour and 7 hours). And, we got good results about sperm quality, Caspase activity, TUNEL positive germ cells and Johnsen’s and Miller’s criteria for measuring testes tissue architecture.

These comments were great; because, we would like to continue this job in offspring. We will happy to use your good experience in this field.

Saeed Shokri, Ph.D.

P.O.Box: 45139-56111Department of Anatomical SciencesFaculty of MedicineZanjan University of Medical Sciences (ZUMS)ZanjanIran

Email: saeedshus@zums.ac.ir
